# Statistical and machine learning approaches to predict the necessity for computed tomography in children with mild traumatic brain injury

**DOI:** 10.1371/journal.pone.0278562

**Published:** 2023-01-03

**Authors:** Tadashi Miyagawa, Marina Saga, Minami Sasaki, Miyuki Shimizu, Akira Yamaura

**Affiliations:** 1 Department of Pediatric Neurosurgery, Matsudo City General Hospital, Matsudo, Japan; 2 Department of Neurosurgery, Matsudo City General Hospital, Matsudo, Japan; Duke University Medical Center: Duke University Hospital, UNITED STATES

## Abstract

**Background:**

Minor head trauma in children is a common reason for emergency department visits, but the risk of traumatic brain injury (TBI) in those children is very low. Therefore, physicians should consider the indication for computed tomography (CT) to avoid unnecessary radiation exposure to children. The purpose of this study was to statistically assess the differences between control and mild TBI (mTBI). In addition, we also investigate the feasibility of machine learning (ML) to predict the necessity of CT scans in children with mTBI.

**Methods and findings:**

The study enrolled 1100 children under the age of 2 years to assess pre-verbal children. Other inclusion and exclusion criteria were per the PECARN study. Data such as demographics, injury details, medical history, and neurological assessment were used for statistical evaluation and creation of the ML algorithm. The number of children with clinically important TBI (ciTBI), mTBI on CT, and controls was 28, 30, and 1042, respectively. Statistical significance between the control group and clinically significant TBI requiring hospitalization (csTBI: ciTBI+mTBI on CT) was demonstrated for all nonparametric predictors except severity of the injury mechanism. The comparison between the three groups also showed significance for all predictors (p<0.05). This study showed that supervised ML for predicting the need for CT scan can be generated with 95% accuracy. It also revealed the significance of each predictor in the decision tree, especially the "days of life."

**Conclusions:**

These results confirm the role and importance of each of the predictors mentioned in the PECARN study and show that ML could discriminate between children with csTBI and the control group.

## Introduction

Head trauma in children is one of the most mundane for emergency room (ER) visits [1–7], leading to the most common indication for computed tomography (CT) imaging in children [8, 9]. CT has been the current standard for the immediate diagnosis of intracranial injuries. Most children with head trauma have mild traumatic brain injury (mTBI) [8], and more than 90% of CT scans of these patients show no evidence of injury [10–16], with less than 1% of children requiring acute intervention. Because most pediatric head trauma is mild [8, 10, 13], it is difficult to identify clinically significant intracranial injuries that should be treated immediately or require close observation. Nevertheless, CT has been the first choice for children with head trauma in an ER [10–19].

These observations raise concerns that many of the CTs performed for this indication unnecessarily expose children to radiation, which is harmful in the long, leading to increased risk of secondary malignancies [20–22]. In particular, in many children, history, physical examination, and observation over a while are sufficient to rule out significant intracranial injury [23–25]. It is important for physicians in the emergency department to decide whether or not to perform CT for children with head trauma. Clinical decision rules such as PECARN have revealed an excellent algorithm to identify the children with clinically-important traumatic brain injury (ciTBI) and prevented many unnecessary head CT scans in children [26–28].

Artificial intelligence (AI) uses computer systems to simulate cognitive abilities to achieve goals. Machine learning (ML) classification is one of the domains of AI that enables an algorithm or classifier to learn patterns in large, complex datasets and produce useful predictive outputs. The number of published ML studies in neurosurgery is increasing [29–33]. Some of them have focused on the application of ML algorithms to support clinical decision-making in neurosurgery [30]. However, no studies have yet been published as to the use of ML to predict the necessity of CT in children with mTBI.

The purpose of this study was to clarify two issues regarding mTBI and the requirement of a CT scan. Firstly, we tried to statistically assess the differences in the predictors in the PECARN study between the control and the children with mTBI. Secondly, we evaluated the feasibility of ML to predict the necessity of CT scans in children with mTBI.

## Materials and methods

### Inclusion and exclusion criteria

This retrospective study enrolled children who visited our hospital or who were admitted between 2010 and 2018. Children under the age of 2 years were enrolled to assess pre-verbal children. The inclusion and exclusion criteria were based on the criteria of the PECARN study [26] except for age and included children who presented within 24 hours of head injury. This study excluded children with penetrating trauma, known brain tumors, pre-existing neurological disease, ventricular shunts, hemorrhagic disease, Glasgow Coma Scale score 13 or less.

### Standardized assessments

The board-certified neurosurgeons recorded patient history, injury mechanism, physical and neurological symptoms before knowing imaging results.

### Outcome measure

Clinically-important TBI (ciTBI) and mTBI on CT were defined based on those in the PECARN study; ciTBI was defined as death from TBI, neurosurgical intervention for TBI, intubation for more than 24 h, or hospital admission of 2 nights or more. Definition of mTBI on CT included intracranial hemorrhage or contusion, cerebral edema, traumatic infarction, diffuse axonal injury, shearing injury, sigmoid sinus thrombosis, midline shift of intracranial contents or signs of brain herniation, diastasis of the skull, pneumocephalus, and skull fracture depressed at least the width of the table of the skull. We defined clinically-significant TBI (csTBI) included ciTBI and mTBI on CT because of requiring at least hospital admission for observation or further treatment.

CT scans were obtained at the clinician’s discretion with helical CT scanners, with radiographic slices separated by 5mm or less. Before the application of the PECARN criteria, criteria for performing CT scans in our hospital were based on physician judgment and caregiver preference, although children with impaired consciousness, a history of LOC, and a history of seizures were of course considered. CT scans were interpreted by site board-certified neurosurgeons.

### Selection of predictors

Risk predictors were described based on those of the PECARN study [26], including gender, the severity of injury mechanism, history of loss of consciousness (LOC), LOC duration, history of vomiting, number of vomiting, acting abnormally per caregivers, Glasgow Coma Scale (GCS), altered mental status, signs of basilar skull fracture, palpable fracture, and scalp hematoma. Age was recorded in days in this study. Injury mechanisms were divided a priori into three categories [26]: severe, moderate, and mild. These predictors except for gender and days of life were categorized as shown in Table 1.

**Table 1:** Categorization of the predictors.

	0	1	2	3	4
severity of injury mechanism	mild	moderate	severe		
Hx of LOC	-	+			
LOC duration	-	<5sec	5-60sec	1-5min	>5min
Hx of vomiting	-	+			
No of vomiting	0	1	2	>2	
acting abnormally	-	+			
GCS	15	14			
altered mental status	-	+			
signs of basilar skull Fx	-	+			
palpable skull Fx	-	+			
scalp hematoma	-	F	T or P	O	

Abbreviations: Hx, history; LOC, loss of consciousness; No, number; GCS, Glasgow Coma Scale; Fx, fracture; F, frontal; T, temporal; P. parietal; O, occipital.

### Data analysis

For a two-group comparison of the control and csTBI, an unpaired t-test and the Mann-Whitney U test were used to determine significance for parametric and non-parametric data, respectively. We also performed a three-group comparison among control, mTBI on CT, and ciTBI. For parametric and non-parametric data, unpaired (between groups) one-factor analysis of variance and multiple comparisons and multiple comparisons by Ryan’s method using the Mann-Whitney U test were applied, respectively. All hypothesis tests were conducted against a 2-sided alternative. P value were considered statistically significant when less than .05.

### Machine learning

Our primary analysis sought to understand the predictive accuracy of a local big-data-driven, machine learning approach based on the previously published clinical decision rules and traditional analytic techniques for classification. A decision tree was selected as the modern machine learning-based model. This study used python version 3.7 and its accompanying packages, implemented from packages such as Scikit-Learn. To predict csTBI based on predictors, we applied supervised ML (sML) using a program written in python. The decision tree method was used for the classification of the children. The accuracy of the algorithm was assessed by calculating the precision. The data for this study were divided into two sets: a training data set and a test data set. The training dataset accounted for 80% of the total data in the evaluation of the predictive model using machine learning. The performance of the predictive models was evaluated using Receiver Operating Characteristic (ROC) curves, specifically Area Under Curve (AUC).

In order to investigate the risk of mTBI (csTBI) at a specific days of life, the outcome of mTBI (csTBI) was plotted against the days of life.

This study complies with the standards of the Declaration of Helsinki and the current ethical guidelines. The study also was approved by the institutional ethics board and by the IRB. Verbal consent was obtained from the caregivers for using the data.

## Results

Table 2 showed the demographic characteristics in control, mTBI on CT, ciTBI, and csTBI. The female ratio and days of life in the control group were significantly higher than in mTBI on CT, ciTBI, and csTBI, respectively (Tables 3, 4).

**Table 2:** Demographic features in control, mTBI on CT, ciTBI and csTBI.

	control	mTBI on CT	ciTBI	csTBI
Number	1042	30	28	58
Gender (female ratio)	0.44	0.30	0.32	0.31
Days of life	392.1±201.7	230.7±141.1	292.1±206.2	260.3±176.7

Abbreviations: mTBI, mild traumatic brain injury, history; CT, computed tomography; ciTBI, clinically important traumatic brain injury; csTBI, clinically significant traumatic brain injury.

**Table 3:** Two-group comparison of the predictors.

	csTBI n = 58	control n = 1042	p
**gender**			0.05
**male**	40	581	
**female**	18	461	
**Days of life**	260.3±176.7	392.2±201.8	<0.01
**severity of injury mechanism**			0.06
**mild**	17	382	
**moderate**	22	444	
**severe**	19	216	
**Hx of LOC**			<0.01
**No**	50	1004	
**yes**	8	38	
**LOC duration**			<0.01
**non**	50	1004	
**<5sec**	3	6	
**5-60sec**	4	16	
**1-5min**	1	12	
**>5min**	0	4	
**Hx of vomiting**			<0.01
**no**	43	919	
**yes**	15	123	
**No of vomiting**			<0.01
**0**	43	919	
**1**	6	65	
**2**	3	12	
**>2**	6	46	
**acting abnormally**			
**no**	34	944	<0.01
**yes**	24	98	
**GCS**			
**15**	34	948	<0.01
**14**	24	94	
**altered mental status**			
**no**	40	1002	<0.01
**yes**	18	40	
**signs of basilar skull Fx**			
**no**	57	1042	<0.01
**yes**	1	0	
**palpable skull Fx**			
**no**	52	1042	<0.01
**yes**	6	0	
**scalp hematoma**			<0.01
**non**	23	846	
**F**	8	133	
**T or P**	23	23	
**O**	4	40	

**Table 4:** Three-group comparison of the predictors.

	*p* value		
	control vs mTBI on CT	control vs ciTBI	mTBI on CT vs ciTBI
gender	0.121	0.203	0.861
days of life	0.017	<0.001	0.196
severity of injury mechanism	0.166	0.204	0.980
Hx of LOC	0.928	<0.001	0.018
LOC duration	0.921	<0.001	<0.001
Hx ot vomiting	0.014	0.035	0.886
No of vomiting	0.011	0.035	0.816
acting abnormally	<0.001	0.004	0.011
GCS	<0.001	<0.001	0.042
altered mental status	<0.001	<0.001	0.015
signs of basilar skull Fx	<0.001	N/A	N/A
palpable skull Fx	<0.001	<0.001	0.929
scalp hematoma	0.947	<0.001	<0.001

The ratio of CT obtained in all children was 26.0%, those of each group showed 21.9%, 100%, 100% in control, mTBI on CT, and ciTBI, respectively.

### Group comparison

In the two-group comparison between control and csTBI, statistical significance was observed for all non-parametric predictors except for severity of injury mechanism (Table 3). Table 4 also showed the results in three-group comparisons for all parametric and non-parametric predictors. Based on the results, these predictors were divided into four classes (Table 5).

**Table 5:** Classification of the predictors based on the statistical significances.

		Statistical significance		
class		control vs mTBI on CT	control vs ciTBI	mTBI on CT vs ciTBI
I	gender	**-**	**-**	**-**
	severity of injury mechanism	**-**	**-**	**-**
II	days of life	**+**	**+**	**-**
	Hx ot vomiting	**+**	**+**	**-**
	No of vomiting	**+**	**+**	**-**
	palpable skull Fx	**+**	**+**	**-**
III	Hx of LOC	**-**	**+**	**+**
	LOC duration	**-**	**+**	**+**
	scalp hematoma	**-**	**+**	**+**
IV	acting abnormally	**+**	**+**	**+**
	GCS	**+**	**+**	**+**
	altered mental status	**+**	**+**	**+**

Abbreviations: Hx, history; LOC, loss of consciousness; No, number; GCS, Glasgow Coma Scale; Fx, fracture; TBI, traumatic brain injury, history; CT, computed tomography; ciTBI, clinically important traumatic brain injury; mTBI, mild traumatic brain injury; N/A, not applicable.

### Prediction with machine learning

**Fig 1:**
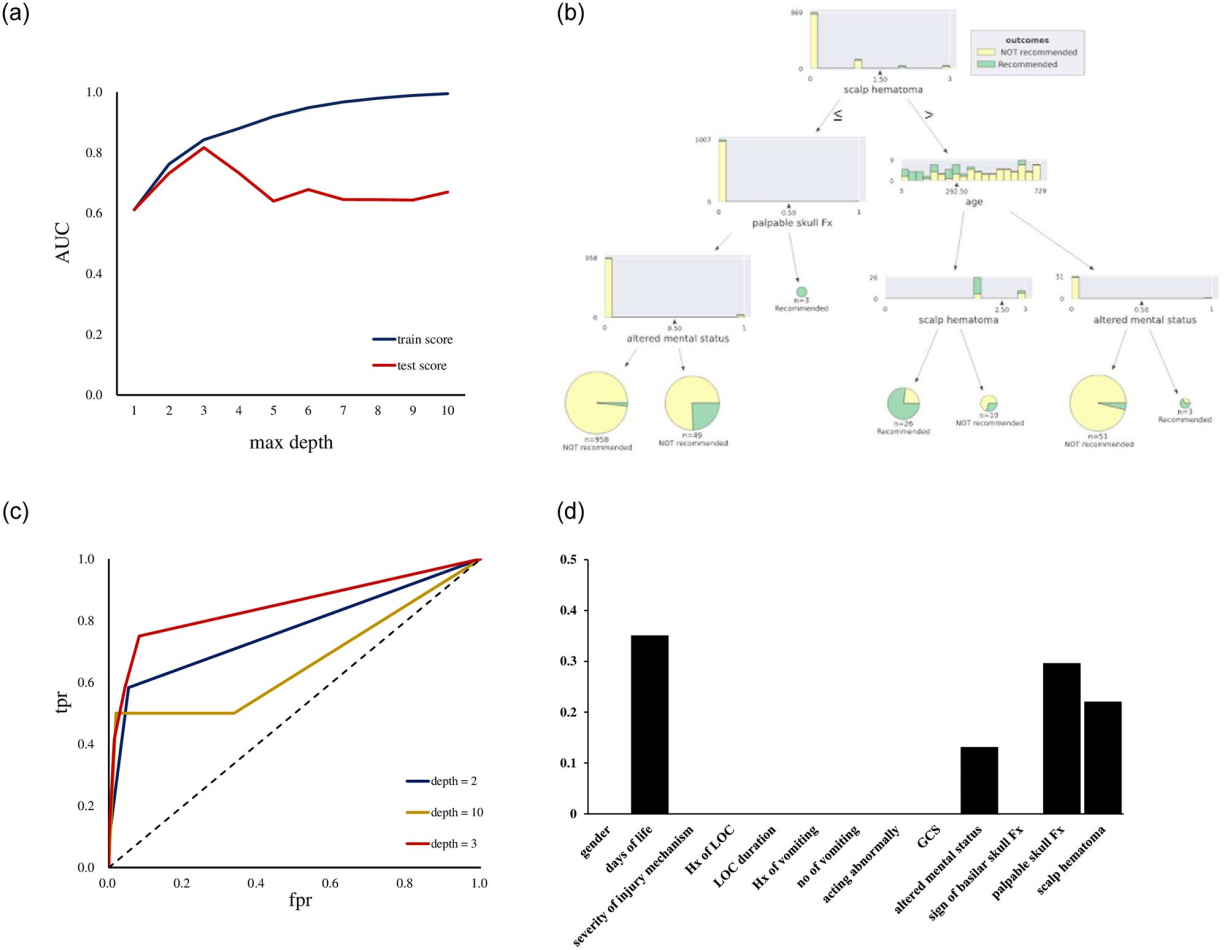
**a**. The area under curve as a function of maximum depth of decision tree, showing peak AUC in test score was shown at the max depth 3, followed by decreasing AUC. Max depth; maximum depth, AUC; area under curve. **b**. The decision tree created by supervised machine learning to predict the necessity of computed tomography of the head for children with minor head trauma. **c**. The relationship between the false positive rate and the true positive rate for max depths of 2, 3, and 10. AUC of the max depth 2,3, 10 were calculated as 0.77, 0.85, 0.66, respectively. fpr; false positive rate, tpr; true positive rate. **d**. The significance of the predictors to create a decision tree, revealing the days of life was most significant. Hx, history; LOC, loss of consciousness; No, number; GCS, Glasgow Coma Scale; Fx, fracture.

**Fig 2:**
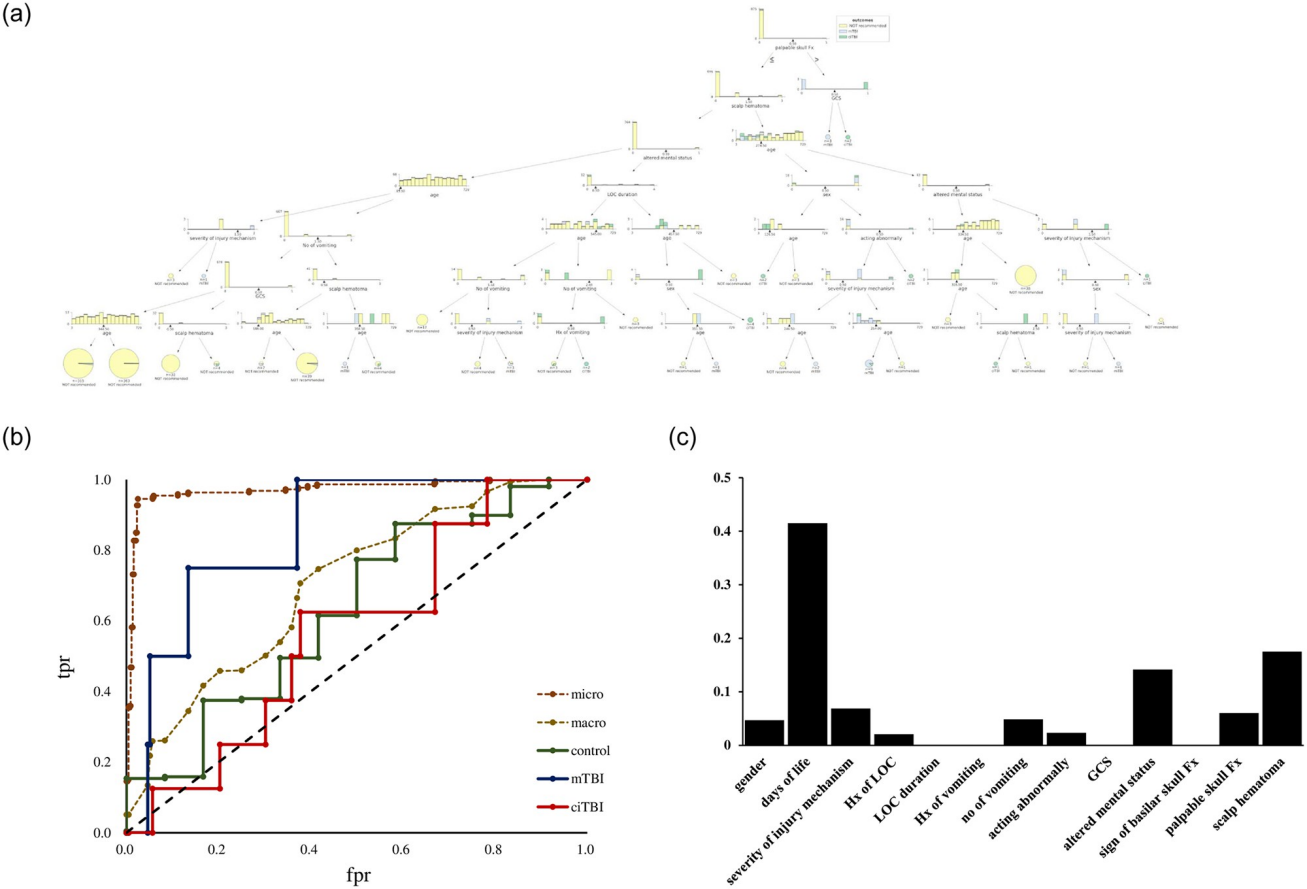
**a**. The decision tree created by supervised machine learning to classify the children as control, mTBI on CT and ciTBI. mTBI; mild traumatic brain injury, CT; computed tomography, ciTBI; clinically important traumatic brain injury. **b**. The relationship between the false positive rate and the true positive rate in control, mTBI on CT and ciTBI. ROC of the control, mTBI on CT, ciTBI were calculated as 0.63, 0.85, 0.57, respectively. fpr; false positive rate, tpr; true positive rate, mTBI; mild traumatic brain injury, CT; computed tomography, ciTBI; clinically important traumatic brain injury. micro; micro-average, macro; macro-average, ROC; receiver operatorating characteristic. **c**. The significance of the predictors to create a decision tree, revealing the days of life was the most significant, followed by scalp hematoma and altered mental status. Hx, history; LOC, loss of consciousness; No, number; GCS, Glasgow Coma Scale; Fx, fracture.

**Fig 3:**
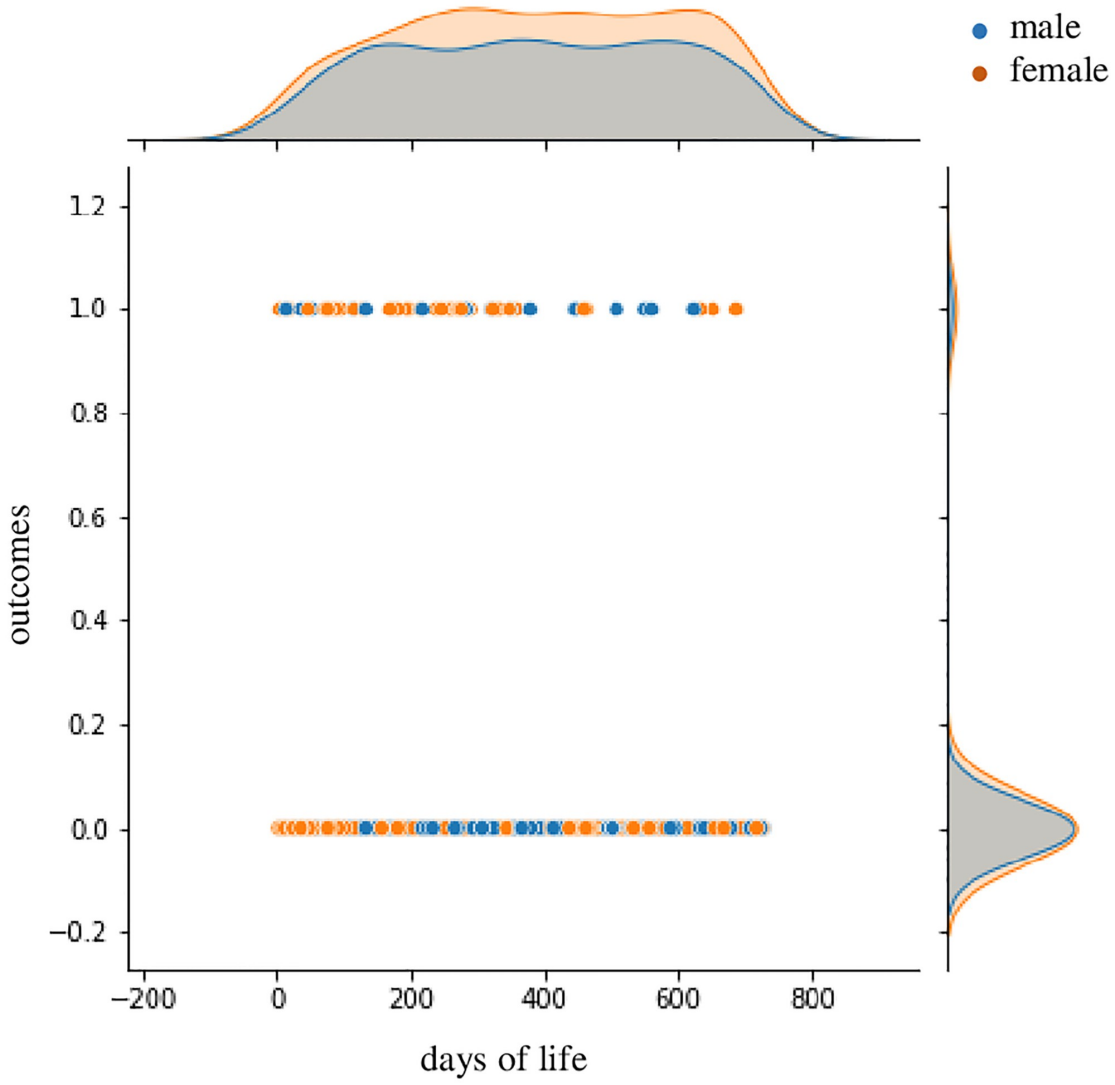
Relationship between the risk of mTBIand days of life, showing no apparent charasteristics. Outcome 0.0 means no risk, outcome 1.0 means mTBI. mTBI, mild traumatic brain injury.

Supervised ML with a decision tree was applied to classify the children into two classes: control children who did not need a CT scan and children with csTBI who needed a CT scan. Fig 1a showed the relationship between the maximum depth (max depth) of the tree and the area under the curve (AUC), revealing that the test data showed a peak AUC at the third depth, followed by a decreasing AUC. Therefore, we created an ML algorithm with this constraint and achieved an accuracy of 0.95 (Fig 1b). Fig 1c shows the relationship between the false positive rate (fpr) and the true positive rate (tpr) for max depths of 2, 3, and 10. In the setting of max depth 3, the accuracy of the training and test data was 0.961 and 0.955, respectively (Table 6). A comparison of the actual and predicted data showed that accuracy, precision, and F1 scores were 0.95, 0.95, and 0.95, respectively. The AUC was 0.85 in the max depth 3. Fig 1d shows the significance of the predictors for creating the decision tree, with the days of life being the most significant. We also created an sML algorithm (Fig 2a) and classified children into three groups: control, mTBI on CT, and ciTBI. Fig 2c showed the relationship between fpr and tpr, indicating that the ROCs for control, mTBI on CT, and ciTBI are 0.63, 0.85, and 0.57, respectively. The accuracy of the training and test data was 0.983 and 0.909, respectively (Table 6). Fig 2b showed the significance of the predictors in classifying the three groups, with the highest number of days of life. Fig 3 demonstrated the risk of mTBI against days of life. There was no apparent correlation between the risk of mTBI and days of life.

**Table 6:** Accuracy and DT score in 2 and 3 classification. DT, decision tree.

	train	test	
**2 classification**			
**accuracy**	0.961	0.955	
**DT score**			0.945
**3 classification**			
**accuracy**	0.983	0.909	
**DT score**			0.950

## Discussion

This study identified two issues regarding the need for CT scans in children with minor head trauma. First, the statistical evaluation on predictors presented in the PECARN study [26] showed a significant difference between control and csTBI, mTBI on CT, or ciTBI, respectively. Secondly, the study showed that sML could be used to predict the necessity of a CT scan of the head with high accuracy for children with mTBI. This study also elucidated the importance of each predictor, especially days of life.

### Demographic features of the children

Table 2 showed the demographic characteristics of children in control, mTBI on CT, ciTBI, and csTBI. In the two-group comparison between control and csTBI, there were statistical differences in days of life although gender showed no difference with p = 0.05 (Table 3). In the three-group comparison, the control group had significantly more days of life than mTBI on CT and ciTBI (Table 4), while there was no difference in days of life between mTBI on CT and ciTBI, or gender.

The CT acquisition rate in this study was 26% of all children. This is lower than the 35% reported in the PECARN study [26]. Meanwhile, the CT acquisition in children with mTBI on CT and ciTBI were 100%, respectively. These findings were better than expected [27, 34–36].

### The comparison regarding the non-parametric predictors

Comparison of the non-parametric predictors between the two groups showed that all predictors except severity of injury mechanism were significant between control and csTBI (Table 3). It means that this study also confirmed most of the predictors in the PECARN study were important to identify children with csTBI. Meanwhile, the non-parametric predictors could be subdivided into four classes to discriminate between the three groups of children: control, mTBI on CT, and ciTBI (Tables 4, 5). Gender and severity of injury mechanism were classified as class I, both of which showed no significance in comparisons between any two of the three groups (Table 5). Class II included days of life, history of vomiting, frequency of vomiting, and palpable skull fractures, which were found to be predictors for clarifying children with mTBI on CT and with ciTBI from control children. Conversely, the class II predictors could not discriminate between children with mTBI on CT and with ciTBI. In addition, history of LOC, LOC duration, and scalp hematoma were classified as class III and showed significance between control and ciTBI and between mTBI on CT and ciTBI, but not between control and mTBI on CT. Taken together, the class II predictors could identify children with csTBI, but it is hard to point out the severity of the head injury. Class III predictors may be used to identify more severe types of traumatic brain injury. All of the class IV predictors relating to consciousness were significant in all of the two-group comparisons among the three groups. In other words, the results suggested that predictors related to consciousness are important when considering the need for CT scans in children with head trauma. The PECARN study showed that six predictors were important: altered mental status, scalp hematoma, LOC, mechanism of injury, palpable skull fracture, and acting normally per parent. In particular, altered mental status and palpable skull fractures were associated with a higher risk of ciTBI. Suggested CT algorithm for children younger than 2 years elucidated that GCS 14 or altered mental status, and palpable skull fracture were the first predictors to pick up the children who require a CT scan [26]. They were classified as II and III in this study, suggesting these results were compatible with those in the PECARN study. In the second branch of the PECARN algorithm, scalp hematomas other than frontal, a history of LOC longer than 5 seconds, severe injury mechanism, and acting abnormally per parent were predictors of excluding children for whom CT was not recommended. These predictors were classified as class III and IV, except for severe injury mechanisms. This suggested that children with minor head trauma requiring CT scans may be picked up by a combination of class II and IV or class III and IV predictors [37–39]. To our best knowledge, this is the first implication that each predictor fulfills its role. The injury mechanism has been previously identified as an independent predictor of TBI [24, 26, 27, 34, 40]. Mechanisms associated with increased risk of TBI in children after blunt injury include high-speed motor vehicle accidents, bicycle-related injury, impact from the high-speed projectile, and fall from a height or downstairs [27, 34, 41]. Nigrovic et al. concluded that children with isolated severe injury mechanism at low risk of ciTBI, and many do not require emergent neuroimaging [42].

### Prediction of the necessity of a CT of the head with sML

With sML using a decision tree method, the children with csTBI could be successfully identified from the control with a prediction accuracy score of 95% (Fig 1b). Fig 1d illustrated the importance of the predictors when creating the decision tree, revealing that days of life was the most important, followed by palpable skull fracture, and scalp hematoma. On the other hand, GCS and signs of basilar skull fracture showed less importance in this decision tree. Because decision trees are powerful and popular prediction methods, this study applied sML with the decision tree method. The final decision tree is very well suited for operational use because it can explain precisely why a particular prediction was made. Decision tree algorithms are known to overfit the training set. It is, therefore, critical to providing information on the performance of the training and test sets separately, as well as information on the parameter tuning of the algorithm such as grid search [43]. The prediction accuracy and AUC were maximized at a maximum depth of 3 when creating the sML algorithm for 2 class classification in this study (Fig 1a and 1c), the training and test achieved a high accuracy of 96.1% and 95.5%, respectively, under these conditions. Accuracy, precision, and F1 score were 0.95, 0.95, 0.95, respectively, which also indicated the effectiveness of the algorithm. We also attempted to use sML to identify children with mTBI on CT or ciTBI from control. Fig 2a showed that a decision tree could be created with sML, with a prediction accuracy score of 95% when applying the max depth 7. The ROC for mTBI on CT was indicated 0.85 as shown in Fig 2c, while the ROC for control and ciTBI showed moderately high. On analysis regarding the contribution of each predictor on the decision tree, days of life was the most significant for identifying the children of each classification (Figs 1d and 2c). Furthermore, day of life with different cutoff values was observed in many branches (Figs 1b and 2a). These findings suggest that days of life may be the most important factor to decide on obtaining CT scans for head trauma in children younger than 2 years of age, and that days of life could be used instead of age in general clinical decision rules. The days of life was employed in this study because we believe that children have important characteristics about time, especially when small children are the subject of clinical research. For example, a child who is 364 days old is to be 0 years old, and a child who is 365 days old is to be 1 year old, but it is natural to assume that there is no significant difference in terms of development and growth. In addition, Figs 1d and 2c revealed the importance of the predictors, such as scalp hematoma, palpable skull fracture, and altered mental status. These predictors were also key factors to identify the children requiring a CT scan in the PECARN algorithm. In the PECARN study, the prediction rule with normal mental status, no scalp hematoma except frontal, no LOC or LOC for less than 5 seconds, non-severe injury mechanism, no palpable skull fracture, and acting normally per caregivers had a negative predictive value of 100% and sensitivity of 100% [26]. To our best knowledge, this is the first expertise analysis that showed the feasibility of the sML to identify children with csTBI from control, and the significance of each predictor, especially days of life. However, Fig 3 could not show a characteristic relationship between the risk of mTBI and days of life.

This study indicated sML could be used to predict the necessity of a head CT regarding childhood mTBI. Although AI-based systems are powerful technologies [44–51], they should not replace the clinical judgment of physicians and medical teams [29–33]. The ideal role of these systems is as a data-driven input to the surgical decision-making process, designed to solve focused problems such as predicting the risk of mTBI in this study.

## Limitation of this study

Regarding demographic characteristics, statistical differences were found between the control group and children with csTBI in two- or three-group comparisons, particularly concerning days of life. This issue may affect the interpretation of the results of this study. Future studies may need better demographic controls. CT scans were not performed on all children because we could not ethically justify exposing children to radiation. As with other decision support tools, these methods provide information to physicians and do not replace their decision-making [52]. In this study, decision tree method was applied to create sML algorithm, further studies using Random Forest, CatBoost and LightGBM, etc may be required for more precise analysis. [53, 54]. Also only the parameters identified in the PECARN study were included in this study, other parameters should be included in the feature to obtain much benefits in performance.

Since the purpose of this study is to determine the feasibility of sML for the problem of CT scans of children with minor head trauma, strict scientific procedures such as under-sampling and bagging were not applied to resolve class imbalances. This issue should be resolved in future studies.

## Conclusion

This study clarified two issues regarding the need for CT scans in children with minor head trauma. First, the evaluation on predictors in the PECARN showed there is a significant difference between control and csTBI, mTBI on CT, or ciTBI, respectively. Secondly, the study showed ML could be used to predict the necessity of a head CT with high accuracy for children with mTBI, and also elucidated the importance of each predictor, especially days of life. These results are substantial for ER physicians because they need to balance radiation exposure with the need to miss serious head trauma in children when they must decide if a child with minor head trauma needs a CT scan.

## References

[1] Marin JR, Weaver MD, Yealy DM, Mannix RC. Trends in visits for traumatic brain injury to emergency departments in the United States. JAMA—J Am Med Assoc. 2014;311(18):1917–1919. doi: 10.1001/jama.2014.3979 24825648

[2] Marin JR, Weaver MD, Barnato AE, Yabes JG, Yealy DM, Roberts MS. Variation in emergency department head computed tomography use for pediatric head trauma. Acad Emerg Med. 2014;21(9):987–995. doi: 10.1111/acem.12458 25269579

[3] Marin JR, Wang L, Winger DG, Mannix RC. Variation in Computed Tomography Imaging for Pediatric Injury-Related Emergency Visits. J Pediatr. 2015;167(4):897–904.e3. doi: 10.1016/j.jpeds.2015.06.052 26233603 PMC4881390

[4] Ide K, Uematsu S, Tetsuhara K, Yoshimura S, Kato T, Kobayashi T. External Validation of the PECARN Head Trauma Prediction Rules in Japan. Acad Emerg Med. 2017;24(3):308–314. doi: 10.1111/acem.13129 27862642

[5] Courtenay LA, Huguet R, González-Aguilera D, Yravedra J. A hybrid geometric morphometric deep learning approach for cut and trampling mark classification. Appl Sci. 2020;10(1). doi: 10.3390/app10010150

[6] Ide K, Uematsu S, Hayano S, et al. Validation of the PECARN head trauma prediction rules in Japan: A multicenter prospective study. Am J Emerg Med. 2019;38(8):1599–1603. doi: 10.1016/j.ajem.2019.158439 31522928

[7] Bertsimas D, Dunn J, Steele DW, Trikalinos TA, Wang Y. Comparison of Machine Learning Optimal Classification Trees with the Pediatric Emergency Care Applied Research Network Head Trauma Decision Rules. JAMA Pediatr. 2019;173(7):648–656. doi: 10.1001/jamapediatrics.2019.1068 31081856 PMC6515573

[8] Faul M., Xu L., Wald M.M. and Coronado VG. Traumatic Brain Injury in the United States: Emergency Department Visits, Hospitalizations and Deaths 2002–2006. Vol 1.; 2010. doi: 10.1002/9781118990810.ch30

[9] Gerber N, Sookraj K, Munnangi S, et al. Impact of the Pediatric Emergency Care Applied Research Network (PECARN) guidelines on emergency department use of head computed tomography at a level I safety-net trauma center. Emerg Radiol. 2019;26(1):45–52. doi: 10.1007/s10140-018-1645-4 30259227

[10] Davis RL, Mullen N, Makela M, Taylor JA, Cohen W, Rivara FP. Cranial Computed Tomography Scans in Children After Minimal Head Injury With Loss of Consciousness. Ann Emerg Med. 1994;24(4):640–645. doi: 10.1016/s0196-0644(94)70273-x 8092590

[11] Lumba-Brown A, Yeates KO, Sarmiento K, et al. Centers for Disease Control and Prevention Guideline on the Diagnosis and Management of Mild Traumatic Brain Injury among Children. JAMA Pediatr. 2018;172(11):e182853–e182853. doi: 10.1001/jamapediatrics.2018.2853 30193284 PMC7006878

[12] Ohana O, Soffer S, Zimlichman E, Klang E. Overuse of CT and MRI in paediatric emergency departments. Br J Radiol. 2018;91(1085). doi: 10.1259/bjr.20170434 29271231 PMC6190788

[13] Homer CJ, Kleinman L. Technical report: minor head injury in children. Pediatrics. 1999;104(6). doi: 10.1542/peds.104.6.e78 10586012

[14] Cheng CY, Pan HY, Li CJ, et al. Physicians’ Risk Tolerance and Head Computed Tomography Use for Pediatric Patients With Minor Head Injury. Pediatr Emerg Care. 2021;37(3):e129–e135. doi: 10.1097/PEC.0000000000001540 29847541 PMC7938907

[15] Schunk JE, Rodgerson JD, Woodward GA. The utility of head computed tomographic scanning in pediatric patients with normal neurologic examination in the emergency department. Pediatr Emerg Care. 1996;12(3):160–165. doi: 10.1097/00006565-199606000-00004 8806136

[16] Zou L, Li H, Jiang Z, et al. Modified decision-making rule supported by scheduled telephone follow-up reduces head computed tomography utilization in children with mild traumatic brain injury: A cohort study. Medicine (Baltimore). 2020;99(18):e20088. doi: 10.1097/MD.0000000000020088 32358394 PMC7440140

[17] Larson DB, Johnson LW, Schnell BM, Goske MJ, Salisbury SR, Forman HP. Rising use of CT in child visits to the emergency department in the United States, 1995–2008. Radiology. 2011;259(3):793–801. doi: 10.1148/radiol.11101939 21467249

[18] Larson DB, Johnson LW, Schnell BM, Salisbury SR, Forman HP. National trends in CT use in the emergency department: 1995–2007. Radiology. 2011;258(1):164–173. doi: 10.1148/radiol.10100640 21115875

[19] Blackwell CD, Gorelick M, Holmes JF, Bandyopadhyay S, Kuppermann N. Pediatric Head Trauma: Changes in Use of Computed Tomography in Emergency Departments in the United States Over Time. Ann Emerg Med. 2007;49(3):320–324. doi: 10.1016/j.annemergmed.2006.09.025 17145113

[20] Brenner DJ, Hall EJ. Computed Tomography—An Increasing Source of Radiation Exposure. N Engl J Med. 2007;357(22):2277–2284. doi: 10.1056/NEJMra072149 18046031

[21] Brenner DJ. Estimating cancer risks from pediatric CT: Going from the qualitative to the quantitative. Pediatr Radiol. 2002;32(4):228–231. doi: 10.1007/s00247-002-0671-1 11956700

[22] Brenner DJ, Elliston CD, Hall EJ, Berdon WE. Estimated risks of radiation-induced fatal cancer from pediatric CT. Am J Roentgenol. 2001;176(2):289–296. doi: 10.2214/ajr.176.2.1760289 11159059

[23] Nigrovic LE, Schunk JE, Foerster A, et al. The effect of observation on cranial computed tomography utilization for children after blunt head trauma. Pediatrics. 2011;127(6):1067–1073. doi: 10.1542/peds.2010-3373 21555498

[24] Nigrovic LE, Kuppermann N. Children with minor blunt head trauma presenting to the emergency department. Pediatrics. 2019;144(6). doi: 10.1542/peds.2019-1495 31771961

[25] Schonfeld D, Fitz BM, Nigrovic LE. Effect of the duration of emergency department observation on computed tomography use in children with minor blunt head trauma. Ann Emerg Med. 2013;62(6):597–603. doi: 10.1016/j.annemergmed.2013.06.020 23910481

[26] Kuppermann N, Holmes JF, Dayan PS, et al. Identification of children at very low risk of clinically-important brain injuries after head trauma: a prospective cohort study. Lancet. 2009;374(9696):1160–1170. doi: 10.1016/S0140-6736(09)61558-0 19758692

[27] Dunning J, Daly JP, Lomas JP, Lecky F, Batchelor J, Mackway-Jones K. Derivation of the children’s head injury algorithm for the prediction of important clinical events decision rule for head injury in children. Arch Dis Child. 2006;91(11):885–891. doi: 10.1136/adc.2005.083980 17056862 PMC2082967

[28] Osmond MH, Klassen TP, Wells GA, et al. CATCH: A clinical decision rule for the use of computed tomography in children with minor head injury. Cmaj. 2010;182(4):341–348. doi: 10.1503/cmaj.091421 20142371 PMC2831681

[29] Buchlak QD, Esmaili N, Leveque JC, et al. Machine learning applications to clinical decision support in neurosurgery: an artificial intelligence augmented systematic review. Neurosurg Rev. 2020;43(5):1235–1253. doi: 10.1007/s10143-019-01163-8 31422572

[30] Miyagawa T, Sasaki M, Yamaura A. Intracranial pressure based decision making: Prediction of suspected increased intracranial pressure with machine learning. PLoS One. 2020;15(10 October):e0240845. doi: 10.1371/journal.pone.0240845 33085690 PMC7577462

[31] Senders JT, Arnaout O, Karhade A V., et al. Natural and artificial intelligence in neurosurgery: A systematic review. Clin Neurosurg. 2018;83(2):181–192. doi: 10.1093/neuros/nyx384 28945910

[32] Senders JT, Zaki MM, Karhade A V., et al. An introduction and overview of machine learning in neurosurgical care. Acta Neurochir (Wien). 2018;160(1):29–38. doi: 10.1007/s00701-017-3385-8 29134342

[33] Celtikci E. A systematic review on machine learning in neurosurgery: The future of decision-making in patient care. Turk Neurosurg. 2018;28(2):167–173. doi: 10.5137/1019-5149.JTN.20059-17.1 28481395

[34] Atabaki SM, Stiell IG, Bazarian JJ, et al. A clinical decision rule for cranial computed tomography in minor pediatric head trauma. Arch Pediatr Adolesc Med. 2008;162(5):439–445. doi: 10.1001/archpedi.162.5.439 18458190

[35] Bouida W, Marghli S, Souissi S, et al. Prediction value of the Canadian CT head rule and the new orleans criteria for positive head CT scan and acute neurosurgical procedures in minor head trauma: A multicenter external validation study. Ann Emerg Med. 2013;61(5):521–527. doi: 10.1016/j.annemergmed.2012.07.016 22921164

[36] Babl FE, Lyttle MD, Bressan S, et al. A prospective observational study to assess the diagnostic accuracy of clinical decision rules for children presenting to emergency departments after head injuries (protocol): The Australasian Paediatric Head Injury Rules Study (APHIRST). BMC Pediatr. 2014;14(1). doi: 10.1186/1471-2431-14-148 24927811 PMC4074143

[37] Babl FE, Borland ML, Phillips N, et al. Accuracy of PECARN, CATCH, and CHALICE head injury decision rules in children: a prospective cohort study. Lancet. 2017;389(10087):2393–2402. doi: 10.1016/S0140-6736(17)30555-X 28410792

[38] Easter JS, Bakes K, Dhaliwal J, Miller M, Caruso E, Haukoos JS. Comparison of PECARN, CATCH, and CHALICE rules for children with minor head injury: A prospective cohort study. Ann Emerg Med. 2014;64(2). doi: 10.1016/j.annemergmed.2014.01.030 24635987 PMC4731042

[39] Gerilmez A, Çalışaneller AT. Compliance with pecarn head injury decision rules in children under two years old. Ulus Travma ve Acil Cerrahi Derg. 2020;26(3):462–468. doi: 10.14744/tjtes.2019.36902 32436970

[40] Khalifa M, Gallego B. Grading and assessment of clinical predictive tools for paediatric head injury: A new evidence-based approach. BMC Emerg Med. 2019;19(1). doi: 10.1186/s12873-019-0249-y 31200643 PMC6570950

[41] Greenberg JK, Yan Y, Carpenter CR, et al. Development and internal validation of a clinical risk score for treating children with mild head trauma and intracranial injury. JAMA Pediatr. 2017;171(4):342–349. doi: 10.1001/jamapediatrics.2016.4520 28192567

[42] Nigrovic LE, Lee LK, Hoyle J, et al. Prevalence of clinically important traumatic brain injuries in children with minor blunt head trauma and isolated severe injury mechanisms. Arch Pediatr Adolesc Med. 2012;166(4):356–361. doi: 10.1001/archpediatrics.2011.1156 22147762

[43] Wong J, Manderson, Abrahamowicz M, et al, Can Hyperparameter Tuning Improve the Performance of a Super Learner?: A Case Study. Epidemiology. 2019;30(4):521–531. doi: 10.1097/EDE.0000000000001027 30985529 PMC6553550

[44] Molaei S, Korley FK, Soroushmehr SMR, et al. A machine learning based approach for identifying traumatic brain injury patients for whom a head CT scan can be avoided. In: Proceedings of the Annual International Conference of the IEEE Engineering in Medicine and Biology Society, EMBS. Vol 2016-October. Institute of Electrical and Electronics Engineers Inc.; 2016:2258–2261. 10.1109/EMBC.2016.7591179 10.1109/EMBC.2016.7591179 28268778

[45] Monteiro M, Newcombe VFJ, Mathieu F, et al. Multiclass semantic segmentation and quantification of traumatic brain injury lesions on head CT using deep learning: an algorithm development and multicentre validation study. Lancet Digit Heal. 2020;2(6):e314–e322. doi: 10.1016/S2589-7500(20)30085-6 33328125

[46] Raj R, Luostarinen T, Pursiainen E, et al. Machine learning-based dynamic mortality prediction after traumatic brain injury. Sci Rep. 2019;9(1):1–13. doi: 10.1038/s41598-019-53889-6 31776366 PMC6881446

[47] Cai Y, Wu S, Zhao W, Li Z, Ji S. Concussion classification via deep learning using whole-brain white matter fiber strains. arXiv. Published online 2017:1–18. 10.1371/journal.pone.0197992 PMC5967816 29795640

[48] Chong SL, Liu N, Barbier S, Ong MEH. Predictive modeling in pediatric traumatic brain injury using machine learning Data analysis, statistics and modelling. BMC Med Res Methodol. 2015;15(1):1–9. doi: 10.1186/s12874-015-0015-0 25886156 PMC4374377

[49] Abujaber A, Fadlalla A, Gammoh D, Abdelrahman H, Mollazehi M, El-Menyar A. Prediction of in-hospital mortality in patients on mechanical ventilation post traumatic brain injury: machine learning approach. BMC Med Inform Decis Mak. 2020;20(1):336. doi: 10.1186/s12911-020-01363-z 33317528 PMC7737377

[50] Vikram A. Designing an AI-Driven System at Scale for Detection of Abusive Head Trauma Using Domain Modeling. Arizona State University; 2020.

[51] Schroder A, Lawrence T, Voets N, et al. A Machine Learning Enhanced Mechanistic Simulation Framework for Functional Deficit Prediction in TBI. Front Bioeng Biotechnol.2021;9:587082. doi: 10.3389/fbioe.2021.587082 33748080 PMC7965982

[52] Reilly BM, Evans AT. Translating clinical research into clinical practice: Impact of using prediction rules to make decisions. Ann Intern Med. 2006;144(3):201–209. doi: 10.7326/0003-4819-144-3-200602070-00009 16461965

[53] Deist TM, Dankers FJWM, Valdes G, et al. Machine learning algorithms for outcome prediction in (chemo)radiotherapy: An empirical comparison of classifiers. Med Phys. 2018;45(7):3449–3459. doi: 10.1002/mp.12967 29763967 PMC6095141

[54] Osman MH, Mohamed RH, Sarhan HM, et al. Machine Learning Model for Predicting Postoperative Survival of Patients with Colorectal Cancer. Cancer Res Treat. 2022;54(2):517–524. doi: 10.4143/crt.2021.206 34126702 PMC9016295

